# Supplementary material for: A cyclic GMP-dependent signalling pathway regulates bacterial phytopathogenesis
Source: EMBO J. 2013 Jul 23;32(18):2430–8. doi: 10.1038/emboj.2013.165 (PMC3770947; doi:10.1038/emboj.2013.165)
Supplement: Supplementary Information [file emboj2013165s1.pdf]

SUPPLEMENTARY INFORMATION

A cyclic GMP-dependent signaling pathway regulates bacterial phytopathogenesis

Shi-Qi An, Ko-Hsin Chin, Melanie Febrer, Yvonne McCarthy, Jauo-Guey Yang, Chung-Liang Liu, David Swarbreck, Jane Rogers, J. Maxwell Dow, Shan-Ho Chou, Robert P. Ryan

Supplementary Materials and Methods

Bacterial strains, plasmids, and culture conditions. *Xanthomonas campestris* pv *campestris* (*Xcc*) strains and culture conditions have been described previously (An *et al*, 2013; Slater *et al*, 2000). Most experiments were carried out in NYGB medium, which comprises 5 g liter^{-1} bacteriological peptone (Oxoid, Basingstoke, U.K.), 3 g liter^{-1} yeast extract (Difco), and 20 g liter^{-1} glycerol. For biofilm formation, *Xcc* was grown in L medium, which comprises 10 g liter-1 bactotryptone (Difco), 5 g liter^{-1} yeast extract, 5 g liter^{-1} sodium chloride, and 1 g liter^{-1} D-glucose. *E. coli* strains were grown in LB medium at 37°C. Other plasmids and strains used are shown in Supplementary Table S4. Where required antibiotics were used at the following concentrations: ampicillin (Amp): 100 μg ml^{-1}; rifampicin (Rif): 50 μg ml^{-1}; gentamycin (Gm): 20 μg ml^{-1}; kanamycin (Km): 20 μg ml^{-1}; tetracycline (Tc):15μg ml^{-1}

General molecular biology methods. Common molecular biological methods such as isolation of plasmid and chromosomal DNA, polymerase chain reaction (PCR), plasmid transformation as well as restriction digestion were carried out using standard protocols (Sambrook *et al*, 1989). PCR products were cleaned using the QIAquick PCR purification kit (Qiagen) and DNA fragments were recovered from agarose gels using QIAquick mini-elute gel purification kit (Qiagen). Primer sequences are available on request.

Transposon mutagenesis and screening. A library of mutants in the *Xcc* 8004 strain was constructed using mariner transposon vector pBT20 as previously described (McCarthy *et al*, 2010). Briefly, 100 ng of transposon-containing plasmid was electroporated into newly prepared electrocompetent *Xcc* cells. Strains carrying the transposon insertion were identified by selection on Gm. The library of mutants was screened for loss of cellular levels of cyclic GMP using 96-well ACE EIA Kit (Cayman™). Briefly, strains were inoculated into microtiter wells containing NYGB medium using a Qpix robot (Genetix™), equal amounts of bacterial cells as adjusted by determination of optical density at 600 nm (OD600) were harvested by centrifugation, and lysed. The cyclic GMP level present in the bacterial lysates for each well was determined using the ACE EIA Kit (Cayman™).

Identification of transposon insertion sites.

Genomic DNA was extracted from transposon mutants of interest as previously described (McCarthy *et al*, 2010). The nucleotide sequence flanking the transposon insertion site was amplified by PCR as previously described (McCarthy *et al*, 2010) using the primers: Rnd1-TnM (5′-GTGAGCGGATAACAATTTCACACAG-3′), and Rnd2-TNm (5′-ACAGGAAACAGGACTCTAGAGG-3′). Amplicons were sequenced using the TnM specific primer: TnMseq (5′-CACCCAGCTTTCTTGTACAC-3′). Sequence data generated were examined with blastn, blastx (http://www.ncbi.nlm.nih.gov/BLAST/) software.

Construction of insertion and deletion mutant strains.

In-frame deletion of selected genes was carried out using pK18*mobsac* as described previously for *rpfG* (An *et al*, 2013; Slater *et al*, 2000). Mutants were also created by the disruption of genes with

the use of the plasmid pK18*mob* as described previously for *Xcc* (Dow *et al*, 2003).

Cellular quantification of cyclic nucleotides.

Cyclic GMP or Cyclic di-GMP was quantified as described previously (Marden *et al*, 2011; Ryan *et al*, 2009) in bacterial strains grown to an OD at 600 nm of 0.8 in NYGB medium.

Site-directed mutagenesis of CYC or cNMP domain from XC_250 or XC_0249.

Site-directed mutagenesis was used to introduce the alanine alterations using mutagenic PCR in a two-step protocol as previously reported (Ryan *et al*, 2010; Ryan *et al*, 2012). In the first round of PCR, two separate reactions were carried out with one of a pair of primers of complementary sequence carrying the desired alteration and the His-tagged domain of interest cloned into pLAFR3 as template. The products of the first round of PCR were used as templates for a second round of PCR (Mutagenic primer sequences will be supplied upon request).

Cyclic nucleotide cyclase assays

Adenylyl and guanylyl cyclase reactions were undertaken in 100 µl reactions containing 0.5 µg μl^{-1} purified protein of interest, 20 mM Tris (pH 8.0), 100 mM NaCl, 0.5 mM ATP or GTP and 10 mM $MnCl_2$ or $MgCl_2$. Reactions were incubated at 25°C, stopped by heating at 75°C for 10 min and then clarified by centrifugation at 15 000 r.p.m. for 10 min. High-pressure liquid chromatography was used to separate nucleotides as described by (Marden *et al*, 2011; Ryan *et al*, 2010). The identity of nucleotides was confirmed by mass spectrometry.

Cyclic di-GMP cyclase reactions were carried out using a standard enzymatic reaction mixture (total volume, 200 µl) that contained 5 µM enzyme in 50 mM Tris-HCl (pH 7.6), 10 mM $MgCl_2$, 0.5 mM EDTA, and 50 mM NaCl. The reaction was started by the addition of 50 µl of GTP (final concentration, 150 µM) to the pre-warmed reaction mixture and was carried out for 60 min. The mixture was immediately placed in a boiling water bath for 3 min, followed by centrifugation at 15,000 x g for 2 min. The supernatant was filtered through a 0.22µm-pore-size filter and analyzed by high-pressure liquid chromatography (Ryan *et al*, 2006; Ryan *et al*, 2010). The identity of cyclic di-GMP as product was confirmed by mass spectrometry.

Construction of protein expression vectors and protein purification.

PCR fragments coding for the domains from the *Xcc* proteins [XC_0249 or XC_0250] were cloned into pET28a for expression as His-tagged fusions as described previously (Ryan *et al*, 2010; Ryan *et al*, 2012). All the plasmid constructs were confirmed by DNA sequencing. His-tagged proteins were purified by nickel affinity chromatography as described previously (Ryan *et al*, 2010). In all cases protein concentration was assayed using NanoDrop®. Purified proteins were stored at -20°C.

Isothermal titration calorimetry (ITC) experiments.

The association constant (Ka) between wild-type XC_0249 full-length protein and its variants with cyclic GMP or cyclic AMP was measured using an ITC200 calorimeter (MicroCal). Titrations of XC_0249 with cyclic GMP or cyclic AMP were carried out at 25°C in an assay buffer containing 20 mM Tris-Cl (pH 8.0), 80 mM NaCl. Samples of XC_0249 for ITC measurements were dialyzed extensively against the assay buffer overnight. The concentration of wild-type XC_0249 and its variants in the cell was 0.05 mM and that of cyclic GMP in the syringe was 0.75 mM. One µl of cyclic GMP was injected into the cell with a time lag of 180 s between each injection for a total of 30 times. ITC data were analyzed by integrating heat exchange amount after subtracting background dilution heat from the apparent values. Data fitting was based on a one-site binding

model using the commercial package provided (Origin) to obtain the values of Ka, ΔH and ΔS. The ΔG value was derived by calculating using the equation ΔG =ΔH - TΔS. These values were listed in Supplementary Table S2.

RNA extraction and preparation.

Three independent cultures of each selected *Xanthomonas* strain were sub-cultured and grown to logarithmic phase (0.7-0.8 OD_{600}) at 30°C in NYGB broth without selection. 800 µl of RNA protect (Qiagen) was added to 400 µl culture and incubated at room temperature for 5 min. Cell suspensions were centrifuged, the supernatant was discarded, and pellets were stored at −80°C. After thawing, 100 µl TE-lysozyme (400 µg/ml) was added and samples were incubated at room temperature. Total RNA was isolated using the RNeasy Mini Kit (Qiagen) whereby cells were homogenised utilising a 20-gauge needle and syringe. Samples were treated with DNase (Ambion) according to manufacturer's instructions and the removal of DNA contamination was confirmed by PCR.

RNAseq: cDNA library construction and sequencing.

RNA quality was assessed on a Bioanalyser PicoChip (Agilent) and RNA quantity was measured using the RNA assay on QuBit fluorometer (Life Technologies). Ribosomal RNA was depleted with Ribo-Zero™ rRNA Removal Kits for Gram-Negative Bacteria (Epicentre). The percentage of rRNA contamination was checked on a Bioanalyser PicoChip (Agilent).

The rRNA-depleted sample was processed using the Illumina TruSeq RNA v2 sample preparation kit. In brief, the sample was chemically fragmented to ~200nt in length and the cleaved RNA fragments were primed with random hexamers into first strand cDNA using reverse transcriptase and random primers. The RNA template was removed and a replacement strand was synthesised to generate ds cDNA. The ds cDNA was end repaired to remove the overhangs from the fragmentation into blunt ends. A single 'A' nucleotide was added to the 3' ends on the blunt fragments, which is complementary to a 'T' nucleotide on the 3' end of the Illumina adapters. At this stage, adapters containing 6 nt barcodes were used for different samples to allow the pooling of multiple samples together. The resulted barcoded samples were enriched by 10 cycles of PCR to amplify the amount of DNA in the library. The final cDNA libraries were sequenced on an Illumina HiSeq2000 as per manufacturer's instructions.

Computational analysis.

Cluster generation was performed using the Illumina cBot and the cDNA fragments were sequenced on the Illumina HiSeq2000 following a standard protocol. The fluorescent images were processed to sequences using the Pipeline Analysis software 1.8 (Illumina). Raw sequence data obtained in Illumina FASTQ-format were first separated by their barcode sequence by comparing the first 6 bases with the expected barcode sequences. Successfully detected barcodes were removed from the sequence leaving reads of 30 nt in length, while reads containing no recognisable barcode sequence were discarded.

Read mapping, annotation and quantification of transcript levels

Reads for each sample were aligned to the *Xanthomonas campestris* pv *campestris* 8004 NC_007086 assembly (Integrated Microbial Genomes (IMG) database, taxon object ID 637000343) using Bowtie version 0.12.7 with default parameters. Transcript abundance was determined for the gene models annotated in the IMG *Xanthomonas campestris* pv *campestris* 8004 genome release IMG/W 2.0 using the Bowtie RNA-Seq BAM alignments for each of the

samples. To estimate the level of transcription for each gene, the number of reads that mapped within each annotated coding sequence (CDS) was determined.

Analysis of differential expression

Differential expression was assessed using Cufflinks (1). Cufflinks reports an expression value for each transcript and gene in Fragments Per Kilobase of exon model per Million mapped fragments (FPKM). A test statistic is also calculated which, after Benjamini-Hochberg correction for multiple-testing was used to determine significant changes in expression between each pair of samples (false discovery rate 0.05).

Quantitative Real-time PCR.

Quantitative RT-PCRs were used to validate RNA-Seq data. Reverse transcription PCR was achieved using a cDNA synthesis kit (Promega) according to the manufacturer's instructions. Specific RT-PCR primers were used to amplify central fragments of approximately 200 bp in length from different genes. Semi-quantitative RT-PCRs were completed using 250 ng/µl cDNA template and PCR Mastermix (Promega) for 24–36 cycles. For qRT-PCRs, quantification of gene expression and melting curve analysis were completed using a LightCycler (Roche) and Platinum SYBR Green qPCR Supermix-UDG (Invitrogen) was used according to manufacturer's instructions. The constitutively expressed housing keeping gene, *16S rRNA* was used as a reference to standardize all samples and replicates.

Virulence assays.

The virulence of *Xcc* to Chinese radish was estimated after bacteria were introduced into the leaves by leaf clipping as previously detailed (An *et al*, 2013; Dow *et al*, 2003; Ryan *et al*, 2007). Bacteria grown overnight in NYGB medium were washed and re-suspended in water to an OD at 600 nm of 0.001. For leaf clipping the last completely expanded leaf was cut with scissors dipped in the bacterial suspensions. Thirty leaves were inoculated for each strain tested. Lesion length was measured 14 days after inoculation. Each strain was tested in at least four separate experiments.

Biofilm Assays.

Biofilm was assessed by attachment to glass and was determined by crystal violet staining. Log-phase-grown bacteria were diluted to OD600 nm = 0.02 in L media broth, and 5 ml was incubated at 30°C for 24 h in 14-ml glass tubes. After gently pouring off the media, bacterial pellicles were washed twice with water and were then stained with 0.1% crystal violet. Tubes were washed and rinsed with water until all unbound dye was removed (Lu *et al*, 2012). Three independent assays were carried out for each strain.

Protein expression and structure analysis.

Cloning, expression, and purification of native and variant proteins

The coding region corresponding to the different XC_0249 constructs were PCR amplified directly from the plant pathogen *Xcc* using the forward primers 5'-CAATGCTGAATTCATGACCTGCCATACCTCCAACGG-3' and 5'-CAATGCTGAATTCATGACCTGCCATACCTCCAACG-3' and the reverse primers 5'-TCCAATGCTCGAGTTAGCTGTTGTCCA ACGGCAATG-3' and 5'-TCCAATGCTCGAGGATCAGCAATTGCTCGTTATT-3', for the XC_0249 full length and XC_0249 cNMP domain constructs, respectively. The PCR products exhibited correct sizes in agarose gel electrophoresis and were further confirmed by DNA sequencing. The PCR products and the pET vector were both treated with XhoI and EcoRI restriction enzymes in 37°C separately. The

treated insert and vector were then mixed and incubated at 16°C overnight for ligation using T4 DNA ligase (Roche).

The final constructs of the XC_0249FL and XC0249NMP(1-150) targets contain a C-terminal His6-tag and expression is under the control of a T7 promoter. Overexpression of the these target proteins was induced by the addition of IPTG to a final concentration of 0.5 mM at 20°C for 20 h. Proteins were then purified by immobilized metal affinity chromatography (IMAC) on a nickel column (Sigma). For crystallization, the XC0249NMP(1-150) target was further purified on a Superdex 75 column (AKTA, Pharmacia).

Crystallization of the XC_0249 cNMP domain bound to cyclic GMP

For crystallization, the native proteins were concentrated to 5 mg/mL in 20 mM Tris-HCl (pH 8.0), 80 mM NaCl using an Amicon Ultra-10 (Millipore). Screening for crystallization conditions was performed by using a sitting-drop vapor diffusion method in 96-well plates (Hampton Research) at 4°C by mixing 0.3μl protein solution with 0.3 μl reagent solution in the presence of 0.8 mM cyclic GMP. Initial screens including the Hampton Clear Strategy Screen 1, the Structure Screens 1 and 2, a systematic PEG-pH screen, and a PEG/Ion screen were carried out using the PHENIX RE crystallization workstation (Rigaku).

Rod-like crystals of XC_0249 cNMP domain/cyclic GMP complex appeared from a reservoir solution comprising 0.2M ammonium acetate, 0.1M sodium acetate, pH4.6 21% w/v PEG 4000 in 2 days. Crystals suitable for diffraction experiments were grown by mixing 1.6 μl protein solution with 0.8 μl reagent solution at 25 °C. The Se-Met labelled XC_0249 cNMP domain/cyclic GMP complex was crystallized under the same conditions.

Data collection and refinement

Crystals were flash-cooled at 100°K under a stream of cold nitrogen. X-ray diffraction data were collected using the National Synchrotron Radiation Research Center (NSRRC) beamline 13C1 in Taiwan. The diffraction of the XC_0249 cNMP/cyclic GMP complex reaches a resolution of 2.1 Å, with a space group of P31. The unit cell dimensions are a=b=48.64, c=118.09，α=β=90°, and γ=120°. The data for the Se-Met-labelled XC_0249cNMP/cyclic GMP crystals were collected in a similar way to a resolution of 3.2 Å.

The collected data were indexed and integrated using the HKL-2000 software. Autosol in the Phenix suite was used to solve the phase by using the single anomalous dispersion (SAD) approach. Autobuild was used to obtain the initial structure of SeMet-XC_0249 cNMP, which was manually adjusted using the MIfit (2010.10) program. The co-ordinate refinement was then iteratively carried out using the CNS program and Phenix_refine subroutine. The final SeMet-XC_0249 cNMP structure was then used as the template for molecular replacement in the Phenix suite to refine against the native diffraction data of the XC_0249 cNMP. An apparent electron density map was observed in the active site region of the 2Fo-Fc map, and a cyclic GMP molecule was successfully fit into this patch with some adjustment. A unbiased Fo-Fc map was generated at a s value of 2.0 to reveal the presence of cyclic GMP in the XC_0249 cNMP crystal structure. The detailed X-ray diffraction data and the refinement results were listed in Supplementary Table S3.

Supplementary References

An SQ, Febrer M, McCarthy Y, Tang DJ, Clissold L, Kaithakotti G, Swarbreck D, Tang JL, Rogers J, Dow JM, Ryan RP (2013) High-resolution transcriptional analysis of the regulatory influence of cell-to-cell signalling reveals novel genes that contribute to *Xanthomonas* phytopathogenesis *Mol Microbiol* (In Press).

Dow JM, Crossman L, Findlay K, He YQ, Feng JX, Tang JL (2003) Biofilm dispersal in *Xanthomonas campestris* is controlled by cell-cell signaling and is required for full virulence to plants. *Proc Nat Acad Sci USA* **100:** 10995-11000.

Leong SA, Ditta GS, Helinski DR (1982) Heme-biosynthesis in Rhizobium - Indentification of a cloned gene coding for delta-aminolevulinic-acid synthetase from *Rhizobium meliloti*. *J Biol Chem* **257:** 8724-8730.

Lu X-H, An S-Q, Tang D-J, McCarthy Y, Tang J-L, Dow JM, Ryan RP (2012) RsmA regulates biofilm formation in *Xanthomonas campestris* through a regulatory network involving cyclic di-GMP and the Clp transcription factor. *PLoS One* **7:** e12.

Marden JN, Dong Q, Roychowdhury S, Berleman JE, Bauer CE (2011) Cyclic GMP controls *Rhodospirillum centenum* cyst development. *Mol Microbiol* **79:** 600-615.

McCarthy Y, Yang L, Twomey KB, Sass A, Tolker-Nielsen T, Mahenthiralingam E, Dow JM, Ryan RP (2010) A sensor kinase recognizing the cell-cell signal BDSF (cis-2-dodecenoic acid) regulates virulence in Burkholderia cenocepacia. *Mol Microbiol* **77:** 1220-1236.

Qian W*, et al.* (2005) Comparative and functional genomic analyses of the pathogenicity of phytopathogen *Xanthomonas campestris* pv. *campestris*. *Genome Research* **15:** 757-767.

Ryan RP, Fouhy Y, Lucey JF, Crossman LC, Spiro S, He YW, Zhang LH, Heeb S, Camara M, Williams P, Dow JM (2006) Cell-cell signaling in Xanthomonas campestris involves an HD-GYP domain protein that functions in cyclic di-GMP turnover. *Proceedings of the National Academy of Sciences of the United States of America* **103:** 6712-6717

Ryan RP, Fouhy Y, Lucey JF, Jiang B-L, He Y-Q, Feng J-X, Tang J-L, Dow JM (2007) Cyclic di-GMP signalling in the virulence and environmental adaptation of *Xanthomonas campestris*. *Mol Microbiol* **63:** 429-442.

Ryan RP, Lucey J, O'Donovan K, McCarthy Y, Yang L, Tolker-Nielsen T, Dow JM (2009) HD-GYP domain proteins regulate biofilm formation and virulence in Pseudomonas aeruginosa. *Environ Microbiol* **11:** 1126-1136.

Ryan RP, McCarthy Y, Andrade M, Farah CS, Armitage JP, Dow JM (2010) Cell-cell signal-dependent dynamic interactions between HD-GYP and GGDEF domain proteins mediate virulence in *Xanthomonas campestris*. *Proc Nat Acad Sci USA* **107:** 5989-5994.

Ryan RP, McCarthy Y, Kiely PA, O'Connor R, Farah CS, Armitage JP, Dow JM (2012) Dynamic complex formation between HD-GYP, GGDEF and PilZ domain proteins regulates motility in *Xanthomonas campestris*. *Mol Microbiol* **86:** 557-567.

Sambrook J, Fritsch EF, Maniatis T (1989) Molecular Cloning: A Laboratory Manual. Cold Spring Harbor Laboratory Press, NY, Vol. 1, 2, 3.

Schafer A*, et al.* (1994) Small mobilizable mutlipurpose cloning vectors derived from the *Escherhia coli* plasmids PK18 and PK19 - Selection of defined delectionsin the chromosome of *Corynebacterium glutamicum*. *Gene* **145:** 69-73.

Slater H, Alvarez-Morales A, Barber CE, Daniels MJ, Dow JM (2000) A two-component system involving an HD-GYP domain protein links cell-cell signalling to pathogenicity gene expression in *Xanthomonas campestris*. *Mole Microbiol* **38:** 986-1003.

Staskawicz B, Dahlbeck D, Keen N, & Napoli C (1987) Molecular characterization of cloned avirulence genes from RACE-0 and RACE-0 of *Pseudomonas sringae* pv. *glycinea. J Bacteriol* **169:** 5789-5794.

Tang DJ*, et al.* (2005) The zinc uptake regulator zur is essential for the full virulence of *Xanthomonas campestris* pv. *campestris*. Mol Plant Microbe Interact **18:** 652-658.

Turner P, Barber C, Daniels M (1985) Evidence for clustered pathogenicity genes in *Xanthomonas campestris* pv *campestris*. *Mol Gen Genet* **199:** 338-343.

Supplementary Figure and Table Legends

Supplementary Figure 1. (A). Amino acid sequence alignment of the cyclase domain of XC_0250 with cyclase domains of proteins from diverse bacteria including the guanylyl cyclase of *Rhodospirillum centenum*. Abbreviations are: MS: *Mycobacterium smegmatis* Mycsm_05238; BJ: *Bradyrhizobium japonicum* BJ6T_75600; BS: *Burkholderia terrae* WQE_40989 and RC: *Rhodospirillum centenum* RC1_3783.Amino acids predicted to be involved in the formation of the active-site of XC_0250 are boxed in red (D41, D71, L73, A150, L157, A161). (B). Contribution of XC_0250 to cyclic GMP levels in *Xcc*. Deletion of *XC_0250* leads to a reduced level of cyclic GMP in *Xcc* that can be restored to wild-type levels by complementation with a clone (pXC_0250) expressing the full-length XC_0250 protein. Alanine or serine substitutions in residues predicted to be critical for enzymatic activity of XC_0250 abolish restoration (D41A, D71A, L73A, A150S, L157A, A161S). Values given are the mean and standard deviation of triplicate measurements (three biological and three technical replicates). (C). The guanylate cyclase activity of XC_0250 is required for biofilm formation. Variants in the CYC domain of XC_0250 that can no longer function as a guanylate cyclase (D41A, D71A) are unable to restore biofilm formation to the *XC_0250* mutant. Values given are the mean and standard deviation of triplicate measurements (three biological and three technical replicates).

Supplementary Figure 2. Domain organization and cyclic GMP binding by XC_0249. (A). Domain analysis using Pfam and SMART (http://smart.embl-heidelberg.de/) shows that XC_0249 comprises a cNMP domain attached to a GGDEF domain. (B). Binding of radiolabelled cyclic GMP by full-length XC_0249, the isolated cNMP and GGDEF domains of XC_0249 and different variants of the full length XC_0249. All reactions contained 50 µM [^{32}P]-cyclic GMP. Data given as counts per minute (cpm) are the means and standard deviation of triplicate measurements (three biological and three technical replicates).

Supplementary Figure 3. Binding of cyclic GMP to variants of the cNMP domain of XC_0249 as measured by isothermal titration calorimetry. Variants F73A, E90A, H98A, N145A and E146A showed considerably reduced affinity for cyclic GMP, consistent with the role of these residues in cyclic GMP binding.

Supplementary Figure 4. The *in vitro* DGC (diguanylate cyclase) activity of XC_0249 is enhanced by the addition of cyclic GMP at 1 µM. The GTP substrate and cyclic di-GMP product were separated by HPLC. (A). A variant in the cNMP of XC_0249 that no longer bound cyclic GMP (F73A) was unresponsive to the addition of cyclic GMP (A). A variant in the cNMP of

XC_0249 that no longer bound cyclic GMP (F73A, E90A) was unresponsive to the addition of cyclic GMP and less effective than the wild-type in restoring biofilm formation to the *XC_0249* mutant (B). Values given are the mean and standard deviation of triplicate measurements (three biological and three technical replicates).

Supplementary Figure 5. Model of the regulation of biofilm formation and virulence in *Xcc* by cyclic GMP and cyclic di-GMP. The guanylyl cyclase coded by *XC_0250* synthesizes cyclic GMP in response to as yet unknown signal. The cNMP-binding domain of XC_0249 binds and is activated by cyclic GMP, resulting in stimulation of diguanylyl cyclase (GGDEF) domain. This results in the modulation of cyclic di-GMP levels and has downstream effects on biofilm formation and virulence gene expression. It is important to note that cyclic GMP may also be perceived by as yet unknown effectors that contribute to the phenotypes described.

Supplementary Figure 6. Amino acid sequence alignment of the XC_0249 and *E. coli* CRP (*Ec*CRP). (A). The determined secondary structural elements for the cNMP are shown above and below the sequence. The cNMP sequence of *Ec*CRP is followed by a helix-turn-helix DNA binding-domain, while that of XC_0249 is followed by a GGDEF domain. Residues essential for binding cyclic GMP and cAMP are highlighted in either green (identical residues) or red (non-identical residues). The two residues N145 and E146 of XC_0249 form strong H-bonds with the guanine base of cyclic GMP and are indicated by a red arrow. (B). The monomer structure of the cNMP domain of XC_0249.

Supplementary Figure 7. Figure S7. A model showing the superimposition of the cNMP domain of XC_0249 (colored in red) and that of *E. coli* CRP (1G6N) (colored in marine). The central αC helices superimpose/fit strongly while the cNMP binding β -barrel domains deviate due to the binding of different cyclic-mononucleotide. (A). An expanded view of these regions show that the cyclic GMP (the carbons colored in magenta) adopts a *syn*-conformation (the G-H8 and ribose-H1' atoms are located in the same side), which is different to the *anti*-conformation exhibited by cyclic AMP (carbons colored in cyan) in the *E. coli* CRP complex structure (B). It is also important to note that strong H-binding occurs between the cyclic GMP G-O6 and G-2NH2 with the side chain atoms of Asn144 in the αC helix and Glu145' in the αC' helix, respectively. In contrast, in the *E. coli* CRP-cAMP complex, the cyclic AMP A-6NH2 binds with the Ser428 in the αC helix and S428' in the αC' helix.

Supplementary Table 1. List of genes differentially expressed in *XC_0249* and *XC_0250* mutant backgrounds compared to wild-type. Significantly differentially expressed genes (fold change ≥ 4) were determined using Cufflinks after Benjamini-Hochberg correction. The fold change is the ratio of mutant fragments per kilobase of exon per million fragments mapped (FPKM) to wild-type FPKM. The genes were ranked by the size of their fold change in expression.

Supplementary Table 2. Binding constants of XC_0249 variants for different nucleotides as determined by isothermal titration calorimetry.

Supplementary Table 3. Statistics of data collection and structural refinement of XC_0249 cNMP domain bound to cyclic GMP complex

Supplementary Table 4. Strains and plasmids used in this study.

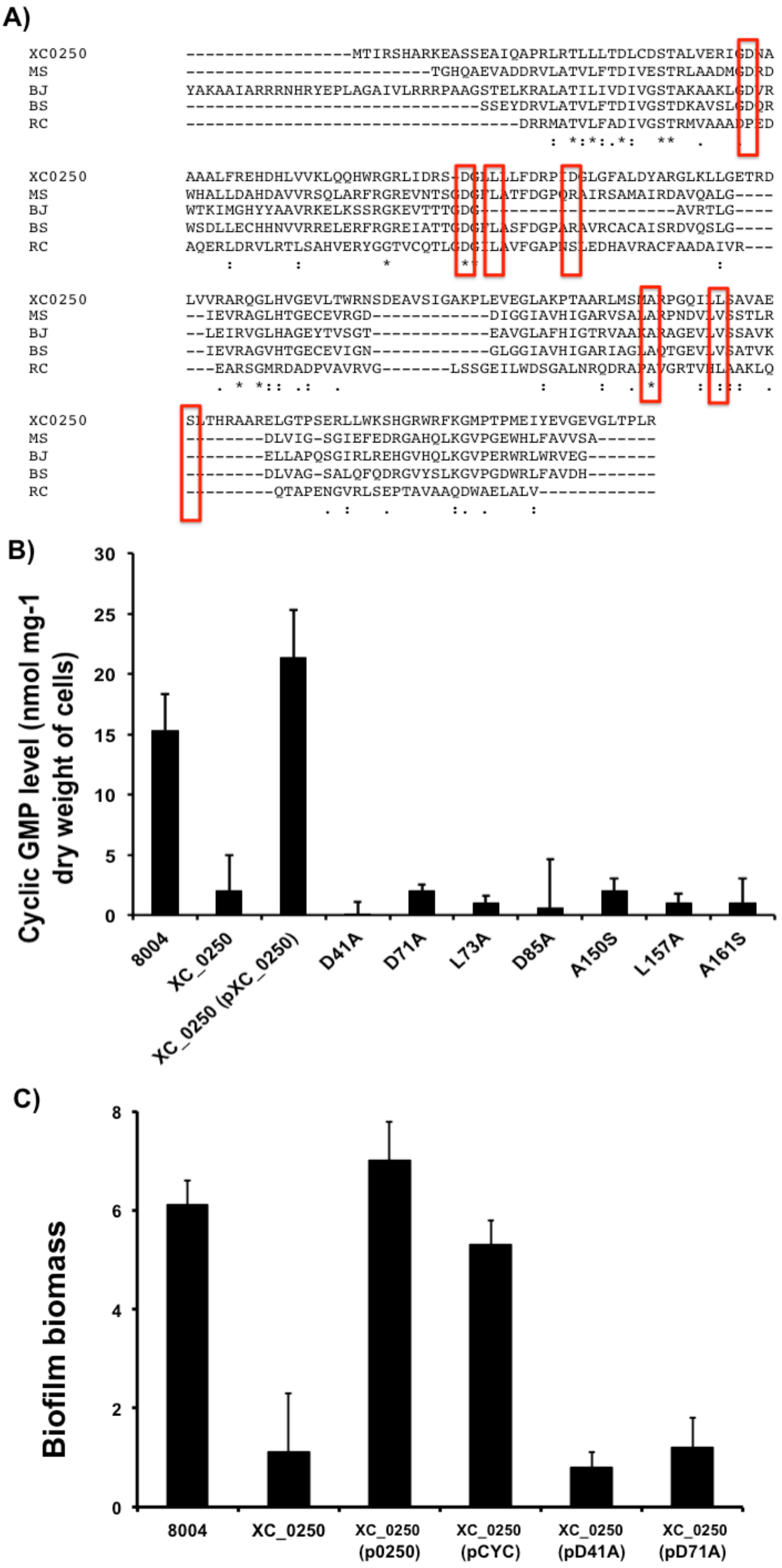
A)
XC0250 ------------------MTIRSHARKEASSEAIQAPRLRTLLLTDLCDSTALVERIGDNA
MS ---------------------------TGHQAEVADDRVLATVLFTDIVESTRLAADMGDRD
BJ YAKAAIARRRNHRYEPLAGAIVLRRRPAAGSTELKRALATILIVDIVGSTAKAAKLGDVR
BS -------------------------------SSEYDRVLATVLFTDIVGSTDKAVSLGDQR
RC -----------------------------------DRRMATVLFADIVGSTRMVAAADPED
XC0250 AAALFREHDHLVVKLQQHWRGRLIDRS-DGLLLLFDRPIDGLGFALDYARGLKLLGETRD
MS WHALLDAHDAVVRSQLARFRGREVNTSGDGFLATFDGPQRAIRSAMAIRDAVQALG----
BJ WTKIMGHYYAAVRKELKSSRGKEVTTTGDG-------------------AVRTLG----
BS WSDLLECHHNVVRRELERFRGREIATTGDGFLASFDGPARAVRCACAISRDVQSLG----
RC AQERLDRVLRTLSAHVERYGGTVCQTLGDGILAVFGAPNSLEDHAVRACFAADAIVR---
XC0250 LVVRARQGLHVGEVLTWRNSDEAVSIGAKPLEVEGLAKPTAARLMSMARPGQILLSAVAE
MS --IEVRAGLHTGECEVRGD------------DIGGIAVHIGARVSALARPNDVLVSSTLR
BJ --LEIRVGLHAGEYTVSGT------------EAVGLAFHIGTRVAAKARAGEVLVSSAVK
BS --IEVRAGVHTGECEVIGN------------GLGGIAVHIGARIAGLAQTGEVLVSATVK
RC ---EARSGMRDADPVAVRVG-------LSSGEILWDSGALNRQDRAPAVGRTVHLAAKLQ
XC0250 SLTHRAARELGTPSERLLWKSHGRWRFKGMPTPMEIYEVGEVGLTPLR
MS ---------DLVIG-SGIEFEDRGAHQLKGVPGEWHLFAVVSA------
BJ ---------ELLAPQSGIRLREHGVHQLKGVPERWRLWRVEG-------
BS ---------DLVAG-SALQFQDRGVYSLKGVPGDWRLFAVDH-------
RC ----------QTAPENGVRLSEPTAVAAQDWAELALV------------
B)
Cyclic GMP level (nmol mg-1 dry weight of cells)
0
5
10
15
20
25
30
8004
XC_0250
XC_0250 (pXC_0250)
D41A
D71A
L73A
D85A
A150S
L157A
A161S
C)
Biofilm biomass
0
2
4
6
8
8004
XC_0250
XC_0250 (p0250)
XC_0250 (pCYC)
XC_0250 (pD41A)
XC_0250 (pD71A)

A)

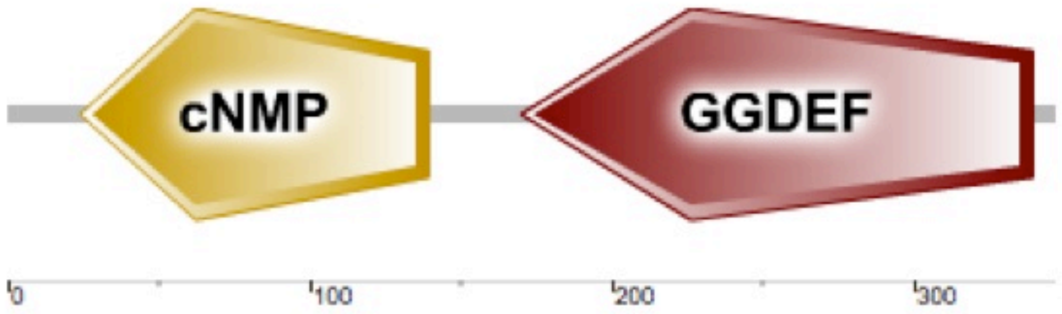
cNMP
GGDEF
0
100
200
300

B)

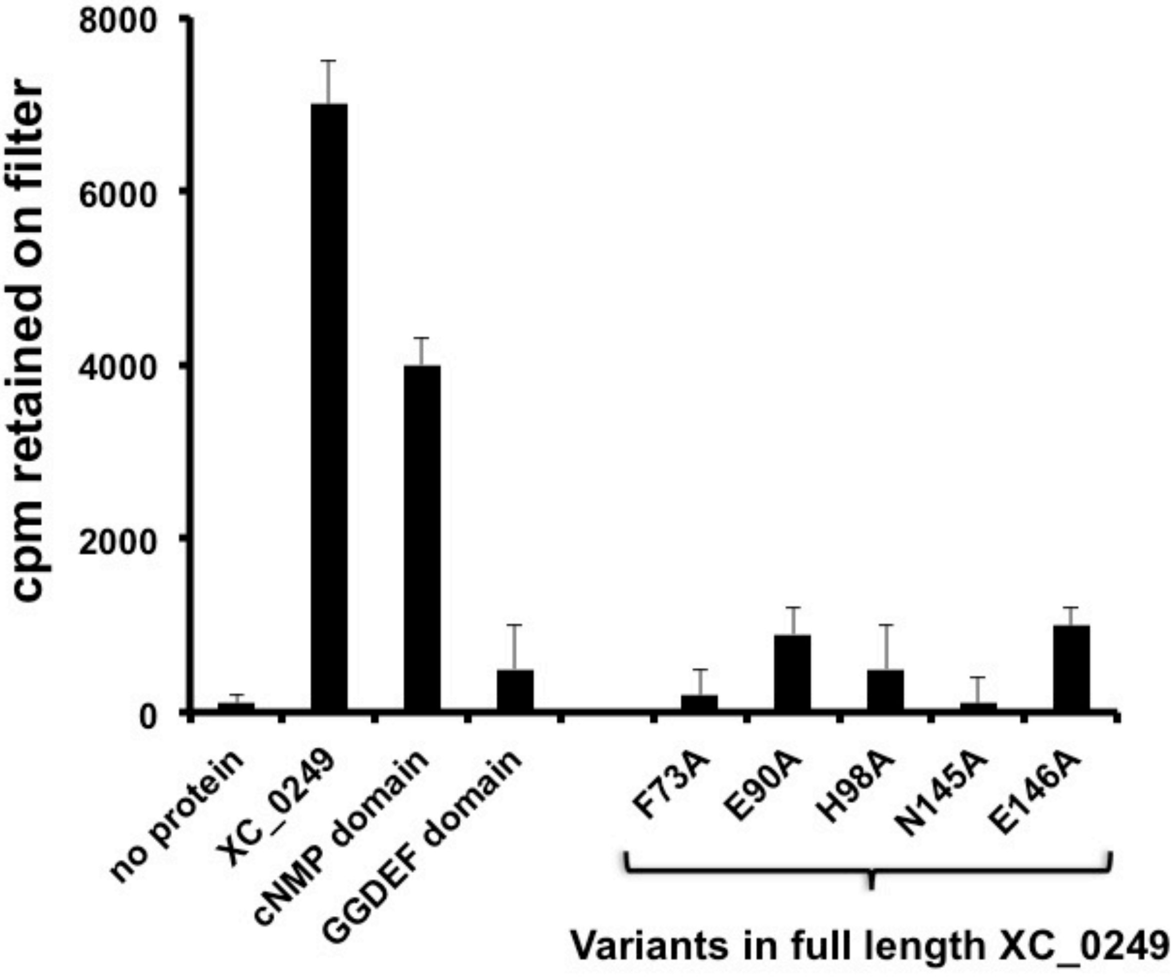
8000
6000
4000
2000
0
cpm retained on filter
no protein
XC_0249
cNMP domain
GGDEF domain
F73A
E90A
H98A
N145A
E146A
Variants in full length XC_0249

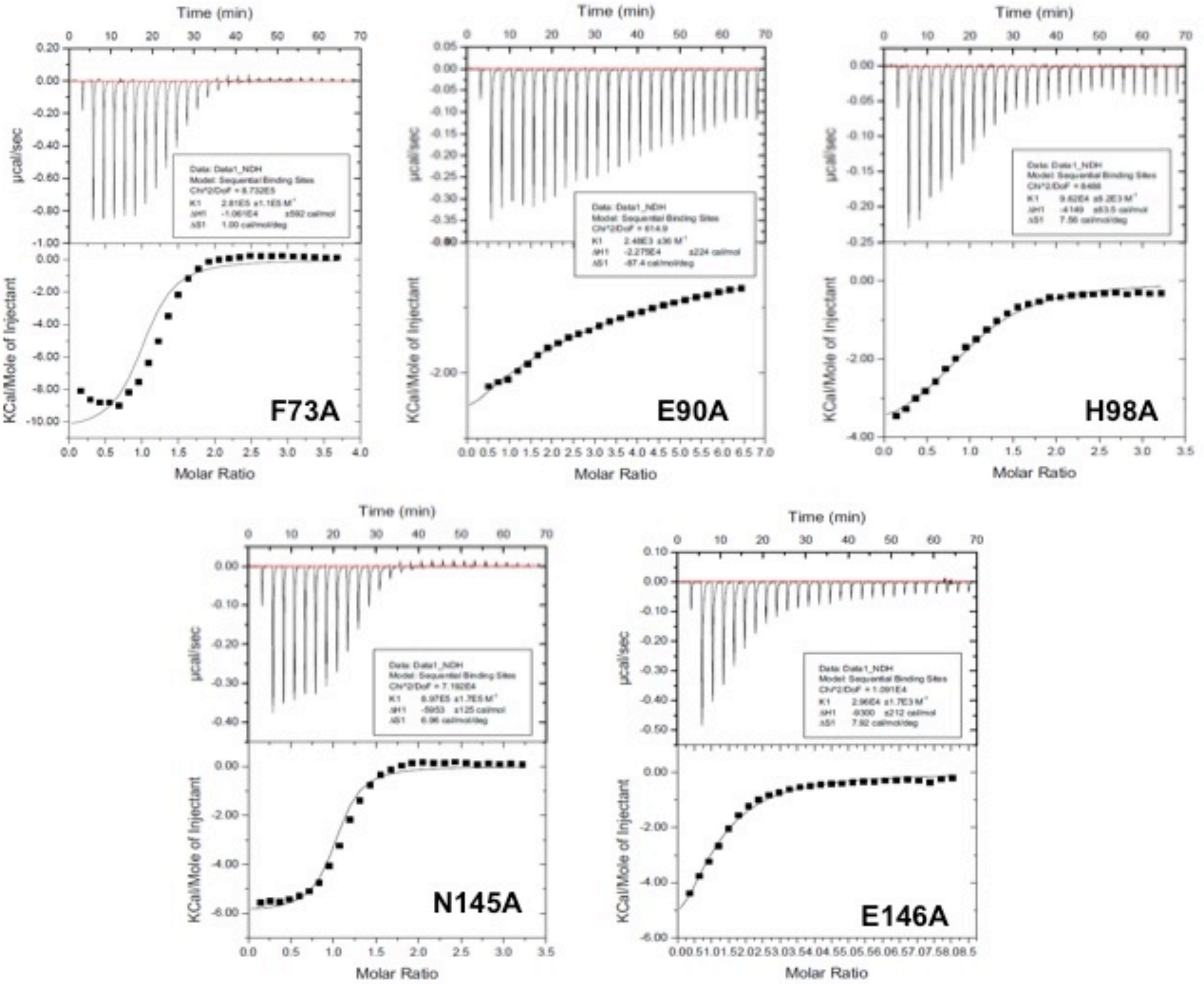
Time (min)
µcal/sec
KCal/Mole of Injectant
Molar Ratio
Data: Data1_NDH
Model: Sequential Binding Sites
F73A
E90A
H98A
N145A
E146A

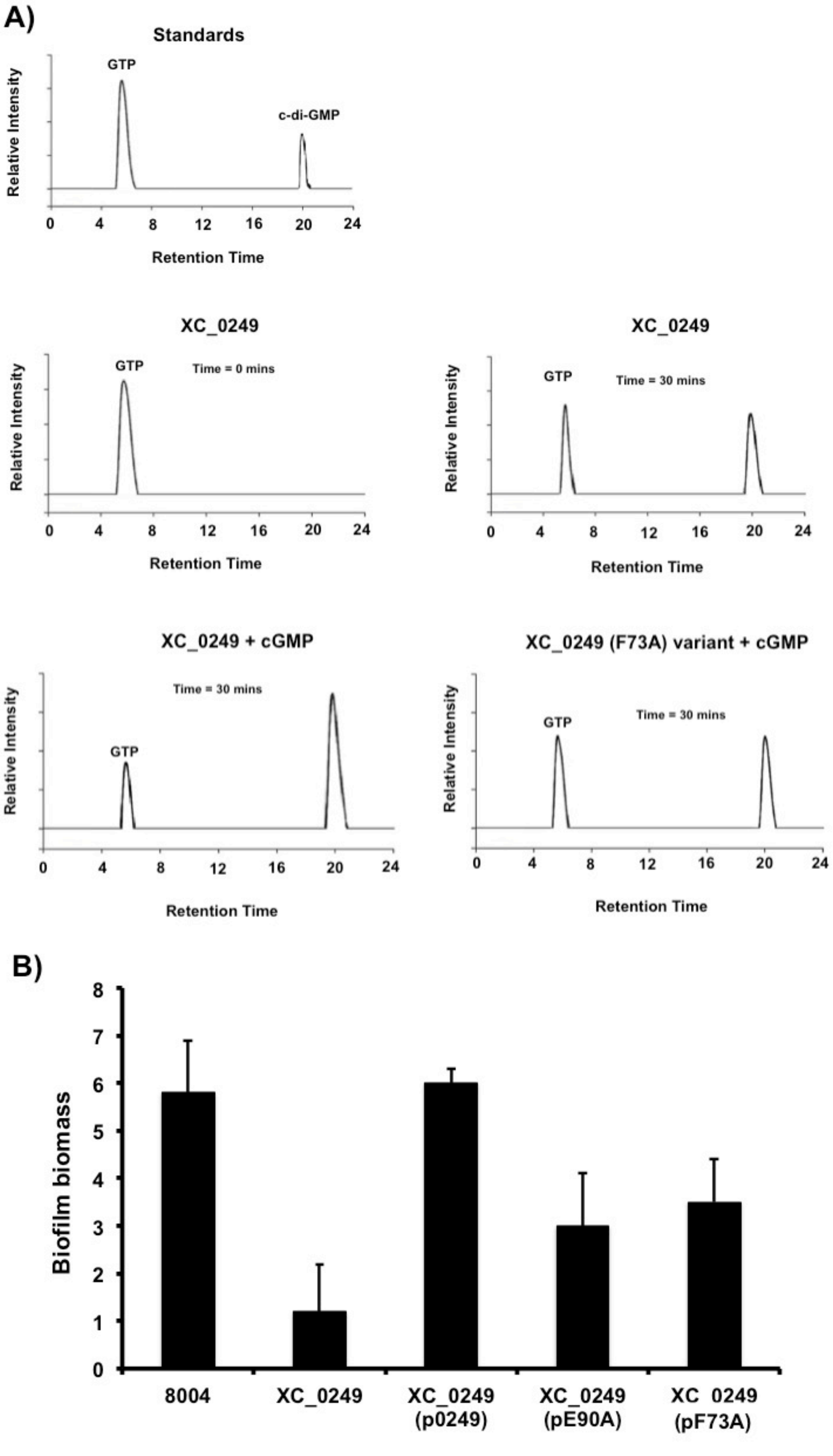

A)
Standards
GTP
c-di-GMP
Relative Intensity
Retention Time
0
4
8
12
16
20
24
XC_0249
Time = 0 mins
XC_0249
Time = 30 mins
XC_0249 + cGMP
XC_0249 (F73A) variant + cGMP
B)
Biofilm biomass
0
1
2
3
4
5
6
7
8
8004
XC_0249
XC_0249 (p0249)
XC_0249 (pE90A)
XC 0249 (pF73A)

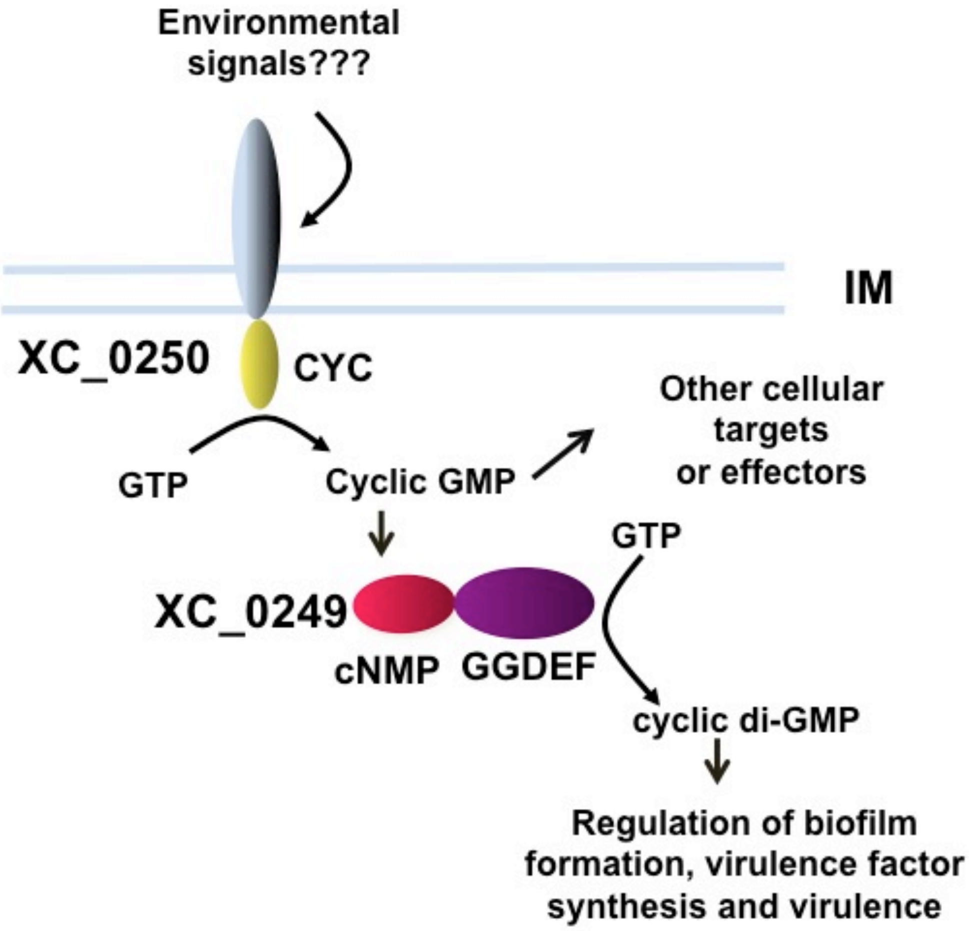
Environmental
signals???
IM
XC_0250
CYC
GTP
Cyclic GMP
Other cellular
targets
or effectors
GTP
XC_0249
cNMP
GGDEF
cyclic di-GMP
Regulation of biofilm
formation, virulence factor
synthesis and virulence

A).

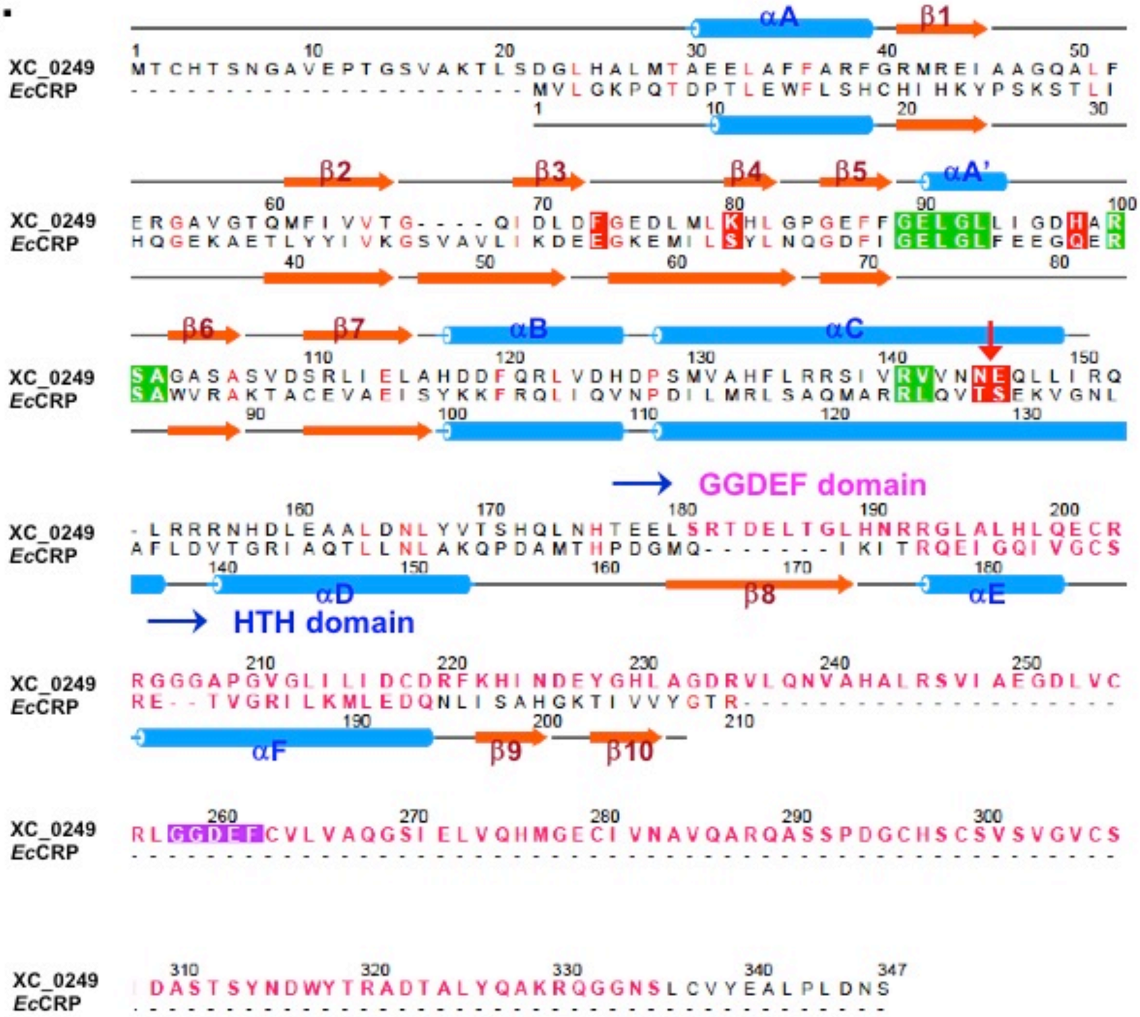
αA β1
XC_0249 MTCHTSNGAVEPTGSVAKTLSDGLHALMTAEELAFFARFGRMREIAAGQALF
EcCRP ---------------------MVLGKPQTDPTLEWFLSHCHIHKYPSKSTLI
β2 β3 β4 β5 αA'
XC_0249 ERGAVGTQMFIVVTG----QIDLDFGEDLMLKHLGPGEFFGELGLLIGDHAR
EcCRP HQGEKAETLYYIVKGSVAVLIKDEEGKEMILSYLNQGDFIGELGLFEEGQER
β6 β7 αB αC
XC_0249 SAGASASVDSRLIELAHDDFQRLVDHDPSMVAHFLRRSIVRVVNNEQLLIRQ
EcCRP SAWVRAKTACEVAEISYKKFRQLIQVNPDILMRLSAQMARRLQVTSEKVGNL
→ GGDEF domain
XC_0249 -LRRRNHDLEAALDNLYVTSHQLNHTEELSRTDELTGLHNRRGLALHLQECR
EcCRP AFLDVTGRIAQTLLNLAKQPDAMTHPDGMQ-------IKITRQEIGQIVGCS
αD β8 αE
→ HTH domain
XC_0249 RGGGAPGVGLILLIDCDRFKHINDEYGHLAGDRVLQNVAHALRSVIAEGDLVC
EcCRP RE--TVGRILKMLEDQNLISAHGKTIVVYGTR--------------------
αF β9 β10
XC_0249 RLGGDEFCVLVAQGSIELVQHMGECIVNAVQARQASSPDGCHSCSVSVGVCS
EcCRP --
XC_0249 IDASTSYNDWYTRADTALYQAKRQGGNSLCVYEALPLDNS
EcCRP --

B).

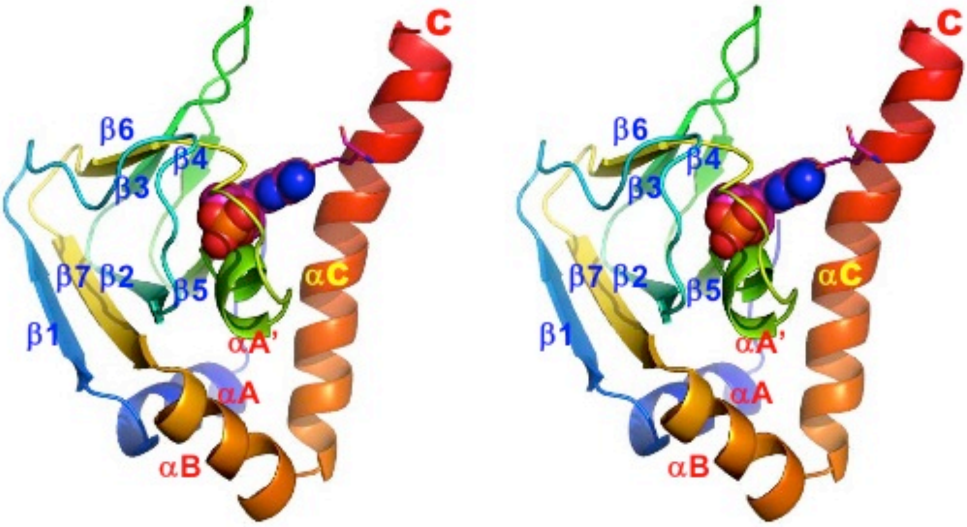
C
β6
β4
β3
β7 β2
β5
αC
β1
αA'
αA
αB

A)

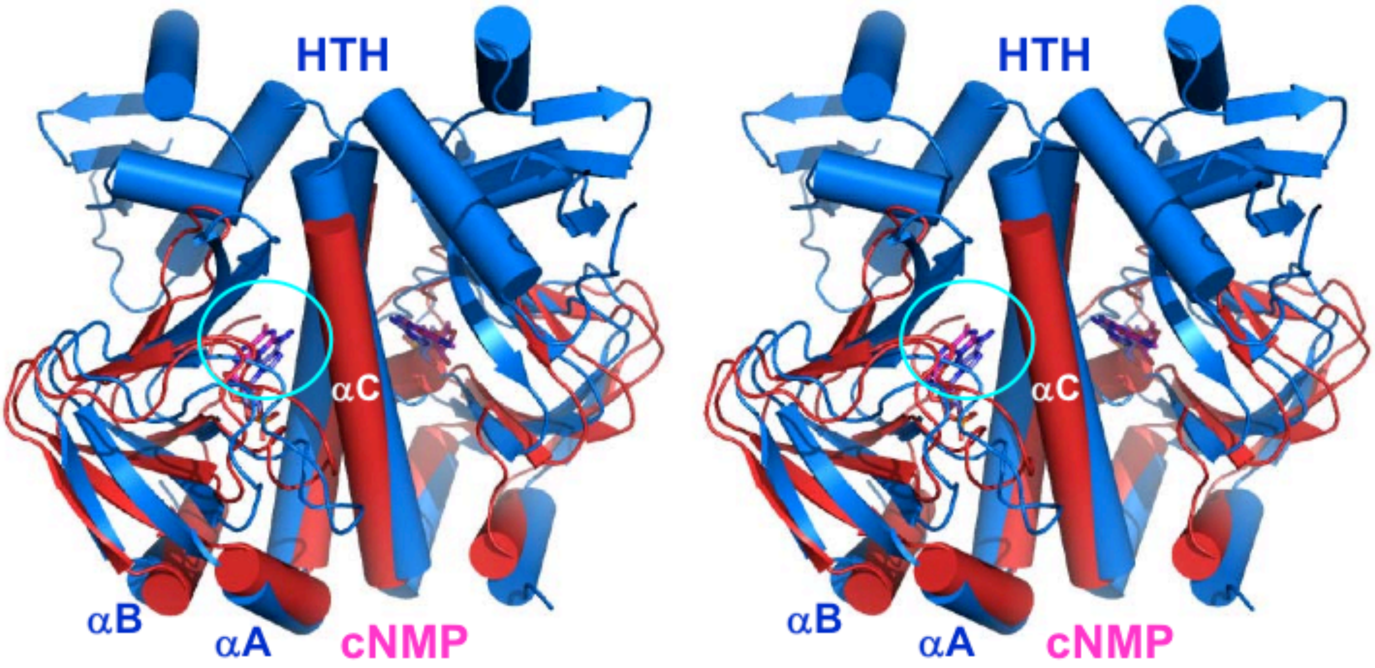
HTH
αC
αB
αA
cNMP
HTH
αC
αB
αA
cNMP

B)

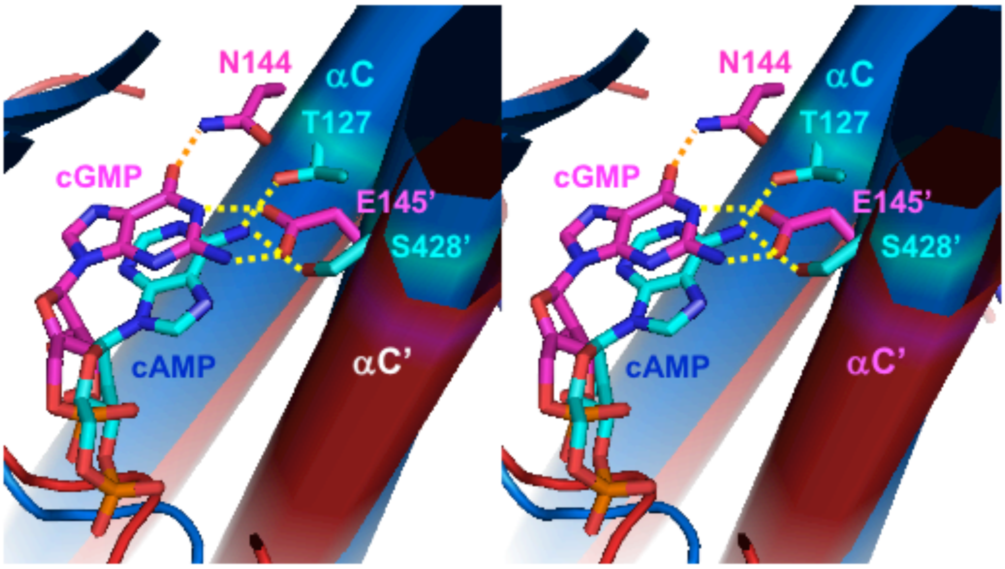
N144
αC
T127
cGMP
E145'
S428'
cAMP
αC'
N144
αC
T127
cGMP
E145'
S428'
cAMP
αC'

Supplementary Table 1. List of genes differentially expressed in *XC_0249* and *XC_0250* mutant backgrounds compared to wild-type. Significantly differentially expressed genes (fold change ≥ 4) were determined using Cufflinks after Benjamini-Hochberg correction. The fold change is the ratio of mutant fragments per kilobase of exon per million fragments mapped (FPKM) to wild-type FPKM. The genes were rankd by the size of their fold change in expression.

**Entry**[a]	**Function**[b]	***XC_0249***[c]	***XC_0250***[c]
XC_0026	cellulase	down	
XC_0027	cellulase	down	
XC_0094	TldD protein	up	up
XC_0117	conserved hypothetical protein	down	down
XC_0129	conserved hypothetical protein		down
XC_0130	Imidazoleglycerol-phosphate synthase		up
XC_0131	conserved hypothetical protein		down
XC_0132	deoxycytidylate deaminase		down
XC_0136	RhsD protein truncated		down
XC_0223	conserved hypothetical protein		down
XC_0224	methyltransferase		down
XC_0225	DNA or RNA helicases of superfamily II		down
XC_0230	conserved hypothetical protein		down
XC_0286	chemotaxis protein	up	up
XC_0323	AlcR transcriptional activator and the CreA global repressor		up
XC_0324	insect-type dehydrogenase		up
XC_0331	AbiD phage protein-like		up
XC_0343	Permeases of the major facilitator superfamily		down
XC_0344	periplasmic serine protease, DO/DeqQ famil		down
XC_0347	lipoprotein		up
XC_0350	putative membrane protein		down
XC_0351	conserved hypothetical protein		down
XC_0412	IS1404 transposase	up	up
XC_0475	conserved hypothetical protein		down
XC_0476	type I restriction-modification system endonuclease		down
XC_0477	putative restriction modification system specificity subunit		down
XC_0478	anticodon nuclease		down

XC_0479	DNA-binding protein		down
XC_0480	type I site-specific deoxyribonuclease		down
XC_0543	TonB-dependent outer membrane receptor	down	down
XC_0544	persistence to inhibition of murein or DNA biosynthesis		up
XC_0585	conserved hypothetical protein		down
XC_0586	putative lipase		down
XC_0637	histidine kinase/response regulator hybrid protein	up	up
XC_0638	chemotaxis protein	down	down
XC_0639	cellulase	down	down
XC_0710	Peptidoglycan-binding domain 1 protein		down
XC_0783	cellulase S	down	down
XC_0817	hypothetical protein		up
XC_0849	TonB-dependent receptor		down
XC_0856	conserved hypothetical protein		down
XC_0857	Lipase (class 3).conserved hypothetical protein		down
XC_0861	conserved hypothetical protein		up
XC_0863	putative lipoprotein		down
XC_0864	VirB6 protein		down
XC_0865	Lipase (class 3)		down
XC_0866	putative exported protein		down
XC_0867	putative lipoprotein		down
XC_0868	VirB6 protein		down
XC_0870	type IV secretion system protein		down
XC_0871	putative secreted protein		down
XC_0914	conserved hypothetical protein		down
XC_0915	TonB-like protein//putative BlaR1 antirepressor		down
XC_0916	transcriptional regulator blaI family		down
XC_0922	reductase		down
XC_0923	transcriptional regulator		up
XC_0924	outer membrane receptor for ferric iron uptake		up
XC_0925	outer membrane receptor for ferric iron uptake		up

XC_0967	HopPtoH;conserved hypothetical protein		up
XC_1004	TonB-dependent receptor	down	down
XC_1018	phage-related integrase		down
XC_1019	conserved hypothetical protein	down	down
XC_1021	Helix-turn-helix XRE-family like proteins		down
XC_1023	conserved hypothetical protein		down
XC_1027	VirB6 protein		down
XC_1028	conserved hypothetical protein		down
XC_1029	conserved hypothetical protein		up
XC_1030	conserved hypothetical protein		down
XC_1036	GGDEFdomain containing protein		up
XC_1037	conserved hypothetical protein		down
XC_1038	conserved hypothetical protein		up
XC_1039	conserved hypothetical protein		down
XC_1040	conserved hypothetical protein		down
XC_1041	conserved hypothetical protein; membrane protein		down
XC_1042	conserved hypothetical protein		up
XC_1057	fimbrial assembly protein		down
XC_1058	pilA; type IV pilus assembly protein PilA		down
XC_1059	pilA; type IV pilus assembly protein PilA		down
XC_1201	RebB protein		up
XC_1213	Xanthomonas outer protein D-XCV/cysteine protease		down
XC_1291	endoproteinase Arg-C	down	down
XC_1292	endoproteinase Arg-C	down	down
XC_1300	quinol oxidase, subunit I	up	up
XC_1301	quinol oxidase, subunit II	up	up
XC_1379	conserved hypothetical protein		down
XC_1380	McrB-related protein		down
XC_1381	McrC, McrBC 5-methylcytosine restriction system component		down
XC_1391	conserved hypothetical protein	down	down
XC_1410	response regulator for chemotaxis	up	up

XC_1411	response regulator	up	up
XC_1413	chemotaxis protein	up	up
XC_1414	chemotaxis histidine protein kinase	up	up
XC_1415	putative anti-anti-sigma factor	up	up
XC_1422	cysteine protease		up
XC_1423	inner membrane protein	up	up
XC_1441	conserved hypothetical protein		up
XC_1442	extracellular serine protease		up
XC_1451	TonB-dependent receptor	down	down
XC_1515	extracellular protease	down	down
XC_1549	hypothetical protein-XCV		down
XC_1621	pre-pilin like leader sequence		down
XC_1622	pre-pilin leader sequence		down
XC_1623	PilW-related protein;Tfp pilus assembly protein		down
XC_1624	PilX related protein		down
XC_1625	Tfp pilus adhesin; type IV pilus assembly protein PilY1		down
XC_1626	type IV pilin		down
XC_1631	putative secreted protein		up
XC_1632	VirB8 protein		down
XC_1633	VirB9 protein		down
XC_1634	VirB10 protein		down
XC_1635	VirB11 protein		down
XC_1636	VirB1 protein		down
XC_1637	VirB2 protein		down
XC_1638	VirB3 protein		down
XC_1639	VirB4 protein		down
XC_1640	hypothetical,ISD1 transposase		down
XC_1719	hypothetical protein		down
XC_1732	Mannosyltransferase OCH1 and related enzymes	up	up
XC_1880	gamma-glutamyl phosphate reductase		down
XC_1945	transport protein		down

XC_2009	Helix-turn-helix motif		down
XC_2011	IS1477 transposase	up	up
XC_2013	sensor kinase		down
XC_2014	conserved hypothetical protein		up
XC_2015	TriD protein		down
XC_2016	VirB6 protein		down
XC_2017	conserved hypothetical protein		down
XC_2018	putative secreted protein-XCV		down
XC_2019	Lipase (class 3).conserved hypothetical protein		down
XC_2044	conserved hypothetical protein		down
XC_2055	putative secreted protein-Xcv		down
XC_2056	conserved hypothetical protein		up
XC_2058	conserved hypothetical protein		down
XC_2087	tannase precursor	down	down
XC_2088	conserved hypothetical protein	down	down
XC_2135	conserved hypothetical protein	up	up
XC_2223	chemotaxis protein	down	down
XC_2224	putative secreted protein	up	up
XC_2230	conserved hypothetical protein	down	down
XC_2231	flagellar protein/negative regulator of flagellin synthesis	down	down
XC_2232	flagellar protein	down	down
XC_2234	flagellar protein/flagellar basal body protein	down	down
XC_2235	flagellar biosynthesis	down	down
XC_2236	flagellar protein/flagellar hook capping protein	down	down
XC_2237	flagellar biosynthesis, flagellar hook protein	down	down
XC_2238	flagellar protein/flagella basal body rod protein	down	down
XC_2239	flagellar biosynthesis	down	down
XC_2240	flagellar L-ring protein	down	down
XC_2241	flagellar protein	down	down
XC_2242	flagellar protein	down	down
XC_2243	flagellar protein	down	down

XC_2244	flagellar protein	down	down
XC_2245	flagellar protein	down	down
XC_2246	flagellar protein	down	down
XC_2247	flagellar protein/flagellin-specific chaperone	down	down
XC_2251	RNA polymerase sigma-54 factor	down	down
XC_2259	flagellar protein	down	down
XC_2260	flagellar protein	down	down
XC_2261	flagellar protein	down	down
XC_2262	flagellar protein	down	down
XC_2264	flagellar FliJ protein	down	down
XC_2265	flagellar protein	down	down
XC_2266	flagellar biosynthesis protein	down	down
XC_2267	flagellar protein	down	down
XC_2269	flagellar protein	down	down
XC_2272	flagellar biosynthesis	down	down
XC_2277	flagellar protein	down	down
XC_2278	flagellar biosynthetic protein FlhA	down	down
XC_2279	flagellar biosynthetic protein,has a GDP-binding motif	down	down
XC_2280	flagellar biosynthesis switch protein	down	down
XC_2281	RNA polymerase sigma factor	down	down
XC_2282	chemotaxis protein	down	down
XC_2283	chemotaxis related protein	down	down
XC_2284	chemotaxis related protein	down	down
XC_2299	chromosome partioning protein	down	down
XC_2300	chemotaxis protein	down	down
XC_2301	putative anti-sigma factor antagonist	down	down
XC_2302	chemotaxis response regulator	down	down
XC_2314	chemotaxis protein	up	up
XC_2318	chemotaxis protein	down	down
XC_2319	conserved hypothetical protein	down	down
XC_2320	chemotaxis protein	down	down

XC_2323	glutamate methylesterase	up	up
XC_2342	regulatory protein rpfI	up	up
XC_2394	IS1477 transposase	up	up
XC_2418	hypothetical protein	down	down
XC_2419	hypothetical protein	down	down
XC_2424	hypothetical protein	down	down
XC_2438	plasmid-related protein	down	down
XC_2456	two-component system sensor protein	up	up
XC_2458	mannan endo-1,4-beta-mannosidase	down	down
XC_2623	IS1477 transposase	up	up
XC_2708	ABC transporter phosphate binding protein	up	up
XC_2830	conserved hypothetical protein	down	down
XC_2848	asparagine synthase B	down	down
XC_2857	proteinU	down	down
XC_2858	pili assembly chaperone	down	down
XC_2859	outer membrane usher protein FasD	down	down
XC_2860	probable lipoprotein signal peptide	down	down
XC_2979	enoyl-CoA hydratase	down	down
XC_2980	acyl-CoA dehydrogenase	down	down
XC_2981	methylmalonate-semialdehyde dehydrogenase	down	down
XC_3214	citrate synthase 2	down	down
XC_3215	carboxyphosphonoenolpyruvate phosphonomutase	down	down
XC_3280	peptidyl-Asp metalloendopeptidase	down	down
XC_3487	alpha-amylase	down	down
XC_3540	conserved hypothetical protein	up	up
XC_3553	endo-1,4-beta-galactanases	up	up
XC_3554	putative membrane protein	up	up
XC_3555	putative glycosyltransferase	up	up
XC_3556	putative secreted protein	up	up
XC_3591	pectate lyase	down	down
XC_3661	conserved hypothetical protein	up	up

XC_3762	cyanide insensitive terminal oxidase	down	down
XC_3763	cyanide insensitive terminal oxidase	down	down
XC_3766	oxidoreductase	up	up
XC_3885	dehydrogenase	down	down
XC_4034	conserved hypothetical protein	down	down
XC_4035	conserved hypothetical protein	down	down
XC_4121	4-oxalomesaconate hydratase	up	up
XC_4152	cytochrome C biogenesis protein	down	down
XC_4153	putative secreted protein	down	down
XC_4291	microcystin dependent protein	down	down
XC_4293	microcystin dependent protein	down	down

a: Annotation according to Qian *et al*, (2005).

b: Predicated function based on best BLAST hits searching the bacterial genome database.

c: Fold change in gene expression in selected mutant compared to wild-type strain - log2 scaled fold change [illegible] expression.

Supplementary Table 2. Binding constants of XC_0249 variants for different nucleotides as determined by isothermal titration calorimetry.

XC_0249 protein and variants	Ka / Kd	Percentage activity#	ΔH Kcal/mol	ΔS cal/mol/deg	ΔG Kcal/mol
WT-cyclic GMP	3.65×10^{6} / 2.74×10^{-7}	100	-9.46	-9.51	-6.63
WT-cyclic AMP	7.13×10^{4} / 1.40×10^{-5}	1.9	-5.44	6.08	-7.25
F73A	2.81×10^{5} / 3.56×10^{-6}	7.6	-10.6	1	-10.9
E90A	2.48×10^{3}/ 4.03×10^{-4}	0.7	-22.75	-87.4	3.3
H98A	9.62×10^{4} / 1.04×10^{-5}	2.6	-4.15	7.56	-6.4
N145A	8.97×10^{5} / 1.11×10^{-6}	24.5	-5.95	6.96	-8.02
E146A	2.96×10^{4} / 3.38×10^{-5}	0.8	-9.3	7.92	-11.66

Supplementary Table 3. Statistics of data collection and structural refinement of XC_0249 cNMP domain bound to cyclic GMP complex.

	**Se-Met substituted**	**Native**
Beamline	NSRRC BL13B1	NSRRC BL13C1
Wavelength (Å)	0.97888, Peak[†]	1.00000
Space group	$P3_1$	$P3_1$
Unit cell parameters	a=b=49.03, c=115.89<br>α=β=90, γ=120	a=b=48.64, c=118.09<br>α=β=90, γ=120
Resolution range (Å)	50-3.21 (3.32-3.21)[*]	30-2.12 (2. 20-2.12)[*]
Total observations	45740	54437
Unique observations	5075	17702
Redundancy	9.0 (7.5)[*]	3.1 (3.0)[*]
Completeness (%)	98.6 (94.2)[*]	99.7 (97.1)[*]
R_{merge}^{Π} (%)	10.2 (30.8)[*]	4.0 (32.0)[*]
I/σ(I)	18.876 (6.212)[*]	18.464 (2.955)[*]
R_{free} test set size (%)		5
Refinement statistics		
$R_{cryst}/R_{free}^{¶}$ (%)		22.54%/26.31%
**Number of atoms**		
Protein residues		260
c-GMP		2
Water		121
**Average B-factors ($Å^2$)**		
Protein residues		38.3
c-GMP		40.1
Water		42.3
**r.m.s deviation from ideal geometry**		
Bonds lengths (Å)		0.0085
Bonds angles (∘)		0.16
**Ramachandran plot** (%)		
Residues in most favorable regions		94.0
Residues in additionally allowed regions		3.9
Residues in generously allowed regions		2.1

* Values in parenthesis are for the outermost shell while the preceding values refer to all data.

¶ R_{free} is the same as R_{cryst} but for 5.0 % of the total reflections chosen at random and omitted from refinement.

† Friedel mates were considered separately as unique reflections in the calculation of these statistics.

Π $R_{merge} = \sum_{hkl}\sum_{i}|\boldsymbol{I}_{i}(hkl) - \langle \boldsymbol{I}(hkl)\rangle| / \sum_{hkl}\sum_{i} \boldsymbol{I}_{i}(hkl)$.

Supplementary Table 4. Strains and plasmids used in this study.

**Strain or plasmid**	**Relevant characteristics**	**Source or Reference**
Escherichia coli		
Escherichia coli DH5α	Cloning strain that supports blue/white screening of colonies,<br>recA1 and endA1	Invitrogen™
Escherichia coli BL21(DE3)	F- ompT gal dcm lon hsdSB(rB- mB-) λ(DE3) pLysS(CmR)	Promega™
**Plasmids and clones**		
pRK2073	Helper plasmid, Sp^R	Leong *et al*, (1982)
pK18*mobkan*	*lacZa*, Km^R	Schäfer *et al*, (1994)
pK18*mobsacB*	*sacB*, *lacZa*, KmR allelic exchange<br>vector	Tang *et al*, (2005)
pLAFR1::Tn5gusA5	Broad-host-range IncP2, cosmid,<br>Km^R, Tc^R	Schäfer *et al*, (1994)
pLAFR3	Broad-host-range IncP2, cosmid, Tc^R	Staskawicz *et al*, (1984)
pPH1JI	Sp^R, Gm^R	Turner *et al*, (1985)
pCR-Blunt-TOPO	Cloning vector for blunt ended PCR products	Invitrogen™
pET-28a	T7 promoter, His tag, lacI, KmR	Novagen™
Xanthomonas campestris		
Xcc 8004	Rif^R	Qian *et al*, (2005)

**Mutants derived from 8004**<br><br>**In-frame unmarked deletion mutants**	**All Rif^R**	
XC_0249	*XC_0249* in frame deletion mutant	This study
XC_0250	*XC_0250* in frame deletion mutant,	This study
**Transposon-insertion mutants**	**All Rif^R, Gm^R**	
XC_0250-MT	As 8004, but *XC_0250*::Tn5gusA5	This study
XC_0250-MT	As 8004, but *XC_0250*::Tn5gusA5	This study
**Site directed variants of the CYC domain of XC_0250**		
D41A	D41A variant introduced by site-directed mutagenesis in the CYC<br><br>domain with a N-terminal His tag cloned into pLAFR3 or without His tag into pET-28a	This study
D71A	D71A variant introduced by site-directed mutagenesis in the CYC<br><br>domain with a N-terminal His tag cloned into pLAFR3 or without His tag into pET-28a	This study
L73A	D73A variant introduced by site-directed mutagenesis in the CYC<br><br>domain with a N-terminal His tag cloned into pLAFR3 or without His tag into pET-28a	This study
L157A	D157A variant introduced by site-directed mutagenesis in the CYC<br><br>domain with a N-terminal His tag cloned into pLAFR3 or without His tag into pET-28a	This study
**Site directed variants of the**		

**cNMP domain of XC_0249**		
F73A	F73A variant introduced by site-directed mutagenesis in the cNMP<br><br>domain with a N-terminal His tag cloned into pLAFR3 or without His tag into pET-28a	This study
E90A	E90A variant introduced by site-directed mutagenesis in the cNMP<br><br>domain with a N-terminal His tag cloned into pLAFR3 or without His tag into pET-28a	This study
H98A	H98A variant introduced by site-directed mutagenesis in the cNMP<br><br>domain with a N-terminal His tag cloned into pLAFR3 or without His tag into pET-28a	This study
N145A	N145A variant introduced by site-directed mutagenesis in the cNMP<br><br>domain with a N-terminal His tag cloned into pLAFR3 or without His tag into pET-28a	This study
E146A	E146A variant introduced by site-directed mutagenesis in the cNMP<br><br>domain with a N-terminal His tag cloned into pLAFR3 or without His tag into pET-28a	This study
